# Barriers to completing oral glucose tolerance testing in women at risk of gestational diabetes

**DOI:** 10.1111/dme.14292

**Published:** 2020-03-18

**Authors:** E. H. Lachmann, R. A. Fox, R. A. Dennison, J. A. Usher‐Smith, C. L. Meek, C. E. Aiken

**Affiliations:** ^1^ School of Clinical Medicine University of Cambridge NIHR Cambridge Comprehensive Biomedical Research Centre Cambridge UK; ^2^ University Department of Obstetrics and Gynaecology University of Cambridge NIHR Cambridge Comprehensive Biomedical Research Centre Cambridge UK; ^3^ The Primary Care Unit Department of Public Health and Primary Care University of Cambridge Cambridge UK; ^4^ Institute of Metabolic Science Cambridge UK; ^5^ Department of Clinical Biochemistry Cambridge University Hospitals Addenbrooke’s Hospital Cambridge UK; ^6^ Wolfson Diabetes and Endocrinology Clinic Cambridge University Hospitals Addenbrooke’s Hospital Cambridge UK; ^7^ Department of Obstetrics and Gynaecology Rosie Hospital Cambridge University Hospitals Cambridge UK; ^8^ Department of Chemistry Peterborough City Hospital Peterborough UK

## Abstract

**Aim:**

Complications of gestational diabetes (GDM) can be mitigated if the diagnosis is recognized. However, some at‐risk women do not complete antenatal diagnostic oral glucose tolerance testing (OGTT). We aimed to understand reasons contributing to non‐completion, particularly to identify modifiable factors.

**Methods:**

Some 1906 women attending a tertiary UK obstetrics centre (2018–2019) were invited for OGTT based on risk‐factor assessment. Demographic information, test results and reasons for non‐completion were collected from the medical record. Logistic regression was used to analyse factors associated with non‐completion.

**Results:**

Some 242 women (12.3%) did not complete at least one OGTT, of whom 32.2% (*n* = 78) never completed testing. In adjusted analysis, any non‐completion was associated with younger maternal age [≤ 30 years; odds ratio (OR) 2.3, 95% confidence interval (CI) 1.6–3.4; *P *< 0.001], Black African ethnicity (OR 2.7, 95% CI 1.2–5.5; *P* = 0.011), lower socio‐economic status (OR 0.9, 95% CI 0.8–1.0; *P* = 0.021) and higher parity (≥ 2; OR 1.8, 95% CI 1.1–2.8; *P* = 0.013). Non‐completion was more likely if testing indications included BMI ≥ 30 kg/m^2^ (OR 1.7, 95% CI 1.1–2.4; *P* = 0.009) or family history of diabetes (OR 2.2, 95% CI 1.5–3.3; *P* < 0.001) and less likely if the indication was an ultrasound finding (OR 0.4, 95% CI 0.2–0.9; *P* = 0.035). We identified a common overlapping cluster of reasons for non‐completion, including inability to tolerate test protocol (21%), social/mental health issues (22%), and difficulty keeping track of multiple antenatal appointments (15%).

**Conclusions:**

There is a need to investigate methods of testing that are easier for high‐risk groups to schedule and tolerate, with fuller explanation of test indications and additional support for vulnerable groups.

## What’s new?

- Gestational diabetes is associated with significant complications if untreated, yet a proportion of at‐risk women invited for antenatal screening do not complete testing. There is a lack of evidence to guide improvements in antenatal screening completion.
- Younger women and those from minority ethnic groups were less likely to complete testing. Key barriers to completion cited by women related to the demands of the testing protocol, ability to attend appointments, and mental health or social issues.
- Modification of testing protocols, increased support for vulnerable groups, and fuller explanation regarding test indications and risk could improve screening rates.

**What’s new?**

- Gestational diabetes is associated with significant complications if untreated, yet a proportion of at‐risk women invited for antenatal screening do not complete testing. There is a lack of evidence to guide improvements in antenatal screening completion.
- Younger women and those from minority ethnic groups were less likely to complete testing. Key barriers to completion cited by women related to the demands of the testing protocol, ability to attend appointments, and mental health or social issues.
- Modification of testing protocols, increased support for vulnerable groups, and fuller explanation regarding test indications and risk could improve screening rates.

## Introduction

Gestational diabetes (GDM) is increasing in prevalence in many maternity populations globally, with current estimates ranging from < 1% to 28% 1. Poorly controlled GDM carries risks for both mother and baby, including macrosomia, birth trauma and emergency Caesarean section 2, 3, 4, 5, 6. The adverse impacts of both maternal hyperglycaemia and accelerated fetal growth can be significantly reduced by available treatment strategies if the diagnosis is made 6, 7. However, GDM diagnosis relies on women attending and completing a relatively complicated standardized testing protocol.

The gold standard test for GDM diagnosis in most contexts is the oral glucose tolerance test (OGTT) 8. Completing an OGTT relies on pregnant women attending a morning appointment in a fasting state, drinking a fixed load of glucose, spending at least two hours in the testing facility, and undergoing multiple blood draws. Evidence suggests that approximately half of women screened experience nausea during the OGTT and a similar proportion find it stressful 9. In the UK, and several other European countries, such testing is offered only to women deemed at high‐risk of GDM 10, 11 due to the expense and inconvenience of the test protocol. Nonetheless, a proportion of women assessed as high‐risk do not complete an OGTT. If women do not attend the appointment, or do not complete the full protocol, this delays or prevents diagnosis and commencement of treatment. Those who never complete the protocol remain at risk of the high complication rates associated with undiagnosed GDM 7.

Previous investigations of barriers to detection and diagnosis of GDM have focused mainly on national healthcare system factors, for example the lack of consensus regarding screening practices 11, 12. There are relatively few studies investigating barriers to antenatal GDM screening from women’s perspectives. Previous work examining attendance for universal GDM screening in Ireland suggests non‐attendance may be influenced by socio‐economic factors and geographical location 13. However, there is little previous evidence concerning completion of risk‐factor based testing. Reasons for non‐completion of antenatal testing in high‐risk women may differ from those in women invited for universal screening. More evidence exists regarding the determinants of engagement with GDM treatment after diagnosis 12 and attendance at postnatal screening. Factors associated with higher postnatal screening rates include older age, lower parity, and higher income or education 14, as well as the proactive contacting of women, and education programmes 15. Key barriers to postnatal screening that have been identified by previous studies include: the demands of an OGTT testing protocol, personal risk perception, lack of education about risk of type 2 diabetes, and competing demands on maternal time 16, 17.

In this study, we aimed to understand which at‐risk sectors of the maternity population are most likely to not complete antenatal testing, and to identify the barriers preventing women from completing an OGTT. This would potentially allow provision of additional support or modification of services to improve completion rates.

## Participants and methods

A cohort of 1906 pregnant women who were consecutively invited to attend for an OGTT at a single tertiary centre in the UK was identified from a contemporaneous database kept to facilitate the clinical follow‐up of results from all OGTTs performed in pregnancy (January to December 2018). During the study period, 5299 women delivered at the study centre, hence ~ 36% of the population were invited for screening. All screening and testing procedures were carried out in line with usual care within the study centre.

Risk factors for GDM were determined at booking (usually performed at 11–16 weeks’ gestation), based on the UK National Institute of Health and Clinical Excellence (NICE) guideline 10. In the study centre, other referrals for OGTT were also made on clinical grounds and all pregnant women were offered a random plasma glucose check at booking with urine dip for glycosuria performed at the same visit.

Indications for testing were categorized as follows for the analysis: BMI ≥ 30 kg/m^2^, high‐risk ethnicity (Black African, Asian or other ethnicities with a high prevalence of diabetes), family history (having a first‐degree relative with type 1 or type 2 diabetes), screening test results (raised random plasma glucose > 7.0 mmol/l or glycosuria), maternal obstetric history [previous macrosomic baby weighing ≥ 4.5 kg or large‐for‐gestational‐age (LGA), previous shoulder dystocia, IVF pregnancy] or medical history (previous bariatric surgery, maternal medication requirements, polycystic ovary syndrome).

Women with any of these indications were referred for a 75‐g 2‐h OGTT, which occurred at 24–28 weeks. Women attended the testing centre following an overnight fast and had a fasting blood sample taken. They drank a standardized 75 g glucose drink provided, then had repeat blood draws at 60 and 120 min. The diagnostic criteria for GDM were based upon modified criteria of the International Association of the Diabetes in Pregnancy Study Groups (IADPSG) (75‐g OGTT 0 h ≥ 5.3; 1 h ≥10.0 mmol/l; 2‐h ≥ 8.5 mmol/l).

We excluded women with a history of GDM in a previous pregnancy, who were seen in clinic soon after booking and commenced on self‐monitoring of capillary blood glucose, as recommended by NICE 10. Women whose pregnancies ended prior to the planned test date were also excluded from the cohort.

Additional OGTTs were performed later in pregnancy on an *ad hoc* basis where clinically indicated; for example, where ultrasound scans later in pregnancy indicated polyhydramnios, high abdominal circumference or LGA. These indications were categorized as scan findings and other *ad hoc* indications for the analysis. In our cohort, the majority (74%) of GDM diagnoses were made between 24 and 28 weeks.

Women who met any of the screening criteria for an OGTT were sent an appointment via post (with instructions regarding how this could be rearranged via telephone if necessary). They also had the option to attend or telephone the phlebotomy clinic directly to arrange an appointment on a convenient day. Full instructions regarding the test protocol were sent to women alongside the appointment confirmation letter.

If women attended the appointment but were unable to complete the full protocol (e.g. due to experiencing vomiting after drinking the glucose load or not following the instructions regarding fasting) this was recorded contemporaneously and testing rebooked.

All test results following planned OGTTs were reviewed by specialist midwives in the obstetric centre whose practice focuses solely on women with or at‐risk of diabetes in pregnancy. Women with positive results were contacted via telephone and asked to attend the next available appointment (usually within a few days) to discuss their diagnosis and initiate treatment. Women with negative results were sent a standard letter via post. Where no result was entered for a planned test, midwives contacted the woman via telephone. During this conversation, all reasons given by the woman for non‐completion of testing were recorded in the woman’s own words by the midwives in the electronic medical record. The appointment was then rebooked at a time and date agreed with the woman. A letter confirming the rebooking and test instructions was sent following the telephone conversation. If it was not possible to contact the woman via telephone after repeated attempts, then this was recorded as ‘no reason given’ and a letter was sent with another appointment.

Detailed data regarding maternal, pregnancy and delivery characteristics were extracted retrospectively from the electronic medical record. Available maternal characteristics included maternal age, maternal BMI (measured at first‐trimester booking), parity (collapsed into categories as 0, 1 and ≥ 2), and ethnicity (collapsed into broad categories: White European, Black African, Asian and other ethnicity). Index of multiple deprivation (IMD) in deciles was derived from postcode data, using 2019 English indices of deprivation data 18. The distance that each woman lived in miles from the hospital was calculated using UK postcode data.

All the reasons given by each woman were extracted verbatim from the electronic medical record and then analysed by the study team to identify common themes. We then categorized reasons into the most common eight themes, with any reasons not falling into these categories classified as ‘other’. Where women gave multiple reasons for not completing testing, all of these were included in the analysis to capture the maximum possible information about barriers to testing.

Three groups were considered in the analysis: women who completed testing at their initial appointment, women with any non‐completed test (all women with one or more non‐completed OGTT) and women who never completed testing (a subset of the former group).

Group‐wise comparisons were carried out using either Student’s *t*‐test or the Mann–Whitney test for numerical data, and Pearson’s chi‐squared test for categorical data. We used logistic regression to model the factors influencing completion of testing for GDM, based on both demographic factors and the indication for testing. Test indication models were adjusted for maternal age and IMD decile, but not for other factors such as BMI or ethnicity because of co‐linearity between demographic characteristics and test indications. Venn diagrams were used to explore and visually represent overlap and clustering of the reasons given by women for non‐completion. Findings were considered statistically significant at an alpha level of 0.05. Power calculations were performed post‐hoc using Monte Carlo simulation, demonstrating that key results are adequately powered. All analyses were conducted using the R statistical software package, version 3.5.1 19.

The study was approved as a service evaluation by the institution (Reasons for non‐attendance at antenatal glucose screening to identify diabetes in pregnancy; Project Record Number 8240).

## Results

### Non‐completion of testing

Of the 1906 women in our cohort, 87.3% (*n* = 1664) of women completed testing at the initial appointment; 12.7% (*n *= 242) of women did not complete at least one OGTT, of whom 32.2% (*n *= 78) never completed testing (Table 1).

**Table 1:** Key characteristics of cohort by attendance status

Category	Completed testing at initial appointment (*n* = 1664; 87%)				
		Any non‐completed test (*n* = 242; 13%)	*P*‐value	Never completed testing (*n* = 78; 4.1%)	*P*‐value
GDM positive					
Yes	169 (10.2)	21 (8.7)		0 (0)	
No	1495 (89.8)	143 (59.1)		0 (0)	
Unknown	0 (0)	78 (32.2)		78 (100)	
Maternal age, years					
≤ 30	480 (28.8)	110 (45.5 )	< 0.001	32 (41.0)	0.178
30–40	985 (59.2)	107 (44.2)	0.271	43 (55.1)	0.567
> 40	192 (11.5)	16 (6.6)	0.338	3 (3.8)	0.072
Unknown	7 (0.4)	9 (3.7)	0.249	0 (0)	0.435
BMI, kg/m^2^					
≤ 25	635 (38.2)	80 (33.1)	0.219	23 (29.5)	0.178
26–35	619 (37.2)	84 (34.7)	0.425	31 (39.7)	0.644
> 35	209 (12.6)	51 (21.1)	0.009	16 (20.5)	0.028
Unknown	201 (12.1)	27 (11.2)	0.632	8 (10.3)	0.329
Ethnicity					
White European	860 (51.7)	110 (45.5)	0.024	33 (42.3)	0.633
Black African	44 (2.6)	12 (5.0)	0.131	3 (3.8)	0.213
Asian	215 (12.9)	22 (9.1)	0.367	4 (5.1)	0.672
Other	353 (21.2)	69 (28.5)	0.031	30 (38.5)	0.050
Unknown	192 (11.5)	29 (12.0)	0.354	8 (10.3)	0.532
Parity					
0	771 (46.3)	90 (37.2)	0.093	26 (33.3)	0.169
1	597 (35.9)	87 (36.0)	0.272	31 (39.7)	0.552
≥ 2	293 (17.6)	57 (23.6)	0.008	20 (25.6)	0.039
Unknown	3 (0.2)	8 (3.3)	0.611	1 (1.3)	0.721
IMD decile	7.3 (6.0–9.0)	6.6 (5.0–8.0)	< 0.001	6.7 (5.0–8.0)	0.042
Distance from hospital (miles)	9.8 (3.2–14.2)	10.6 (3.2–15.1)	0.231	11.0 (3.3–14.9)	0.199

Variables are reported as *n* (%) or mean (IQR). Women who never completed testing are a subset of the group with any non‐completed test. Group‐wise comparisons were carried out using either Student’s *t*‐test or the Mann–Whitney test for numerical data, and Pearson’s chi‐squared test for categorical data. Non‐completion groups were compared to the group who completed testing at initial appointment. IMD, Index of multiple deprivation.

A diagnosis of GDM was equally likely in those who did not complete testing on at least one occasion as in those who completed testing at the first appointment (21 of 242, 8.7% vs. 169 of 1664, 10.2%, *P* = 0.29).

### Demographics of women who do not complete OGTTs

In unadjusted analysis (Table 1), any non‐completion was associated with younger maternal age (≤ 30 years, *P* < 0.001), belonging to an ethnic group other than the main categorizations (*P* = 0.031), having a BMI ≥ 35 kg/m^2^ (*P* = 0.009), having higher levels of deprivation (*P* < 0.001), and having two or more previous children (*P* = 0.008). Any non‐completion was less likely in White European women (*P* = 0.024). A similar set of factors was associated with never completing GDM testing in unadjusted analysis (Table 1).

In adjusted analysis (Table 2), any non‐completed test was associated with younger maternal age [odds ratio (OR) 2.3, 95% confidence interval (CI) 1.6–3.4; *P* < 0.001), being of Black African (OR 2.7, 95% CI 1.2–5.5; *P* = 0.0011) or ‘other’ ethnicity (OR 1.6; 95% CI 1.1–2.3; *P* = 0.0017), and higher parity (OR 1.5, 95% CI 1.0–2.3; *P* = 0.025 for parity 1 and OR 1.8, 95% CI 1.1–2.8; *P* = 0.013 for parity ≥ 2). Women in higher socio‐economic deciles were less likely to have non‐completed tests (OR 0.9, 95% CI 0.8–1.0; *P* = 0.021).

**Table 2:** Characteristics predicting non‐completion of gestational diabetes testing in adjusted analysis

Category	Odds of any non‐completion	*P*‐value	Odds of never completing	*P*‐value
Maternal age, years				
≤ 30	2.3 (1.6–3.4)	< 0.001	1.3 (0.8–2.5)	0.219
30–40	Reference		Reference	
> 40	0.8 (0.4–1.5)	0.572	0.3 (0.0–1.0)	0.104
BMI, kg/m^2^				
≤ 25	Reference		Reference	
26–35	0.9 (0.6–1.3)	0.496	1.3 (0.7–2.6)	0.312
> 35	1.2 (0.7–1.8)	0.464	1.7 (0.8–3.6)	0.162
Ethnicity				
White European	Reference		Reference	
Black African	2.7 (1.2–5.5)	0.011	1.3 (0.2–4.2)	0.839
Asian	0.8 (0.4–1.5)	0.582	0.8 (0.2–1.8)	0.490
Other	1.6 (1.1–2.3)	0.017	2.4 (1.4–4.4)	0.002
Parity				
0	Reference		Reference	
1	1.5 (1.0–2.3)	0.025	1.7 (0.9–3.1)	0.099
≥ 2	1.8 (1.1–2.8)	0.013	2.1 (1.0–4.5)	0.015
IMD decile	0.9 (0.8–1.0)	0.021	0.9 (0.8–1.0)	0.099
Distance from hospital, miles	1.00 (0.9–1.1)	0.532	1.0 (1.0–1.0)	0.564

Values are odds ratios and 95% confidence intervals. Non‐completion groups were compared with the group who completed testing at the initial appointment. Women who never completed testing are a subset of the group with any non‐completed test. Models are adjusted for all other co‐variates listed in the table. IMD, Index of multiple deprivation.

Factors associated with never completing GDM testing in adjusted analysis (Table 2) were belonging to an ethnic group other than the main categorizations (OR 2.4, 95% CI 1.4–4.4; *P* = 0.002) and having two or more previous children (OR 2.1, 95% CI 1.0–4.5; *P* = 0.015).

### Non‐attendance by indication for testing

Some 835 (43.8%) women had multiple indications for an OGTT. In analysis adjusted for all categorized indications plus maternal age and IMD decile (Table 3), there was a higher likelihood of non‐completion in women whose indications for testing included a family history of diabetes (OR 2.2, 95% CI 1.5–3.3; *P* < 0.001) or a high BMI (OR 1.7, 95% CI 1.1–2.4; *P* = 0.009). By contrast, women were less likely to not complete testing if they were recommended to have an OGTT on the basis of scan results (OR 0.4, 95% CI 0.2–0.9; *P* = 0.035). There were no women who never completed testing with scan findings or test results as an indication.

**Table 3:** Likelihood of non‐completing by indication for testing

Indication for testing	Odds of any non‐completion	*P*‐value	Odds of never completing	*P*‐value
BMI ≥ 30 kg/m^2^ (*n* = 610)	1.7 (1.1–2.4)	0.009	1.9 (1.1–3.3)	0.028
High‐risk ethnicity (*n* = 428)	1.4 (0.9–2.1)	0.165	0.8 (0.4–1.7)	0.638
Scan findings (*n* = 200)	0.4 (0.2–0.9)	0.035	Infinity	
Family history (*n* = 452)	2.2 (1.5–3.3)	<0.001	2.8 (1.6–4.9)	< 0.001
Screening test results (*n* = 103)	0.4 (0.1–1.0)	0.110	Infinity	
Maternal obstetric or medical history (*n* = 369)	1.3 (0.8–2.1)	0.342	1.8 (0.8–3.0)	0.131
Multiple pregnancy (*n* = 77)	0.8 (0.3–2.0)	0.721	1.1 (0.2–4.0)	0.864
Other indication (*n* = 29)	0.8 (0.1–3.1)	0.821	1.4 (0.0–7.3)	0.740

Values are odds ratios and 95% confidence intervals. Non‐completion groups were compared with the group who completed testing at initial appointment. Women who never completed testing are a subset of the group with any non‐completed test. Models are adjusted for all other co‐variates listed in table, plus maternal age and Index of multiple deprivation (IMD) decile.

### Reasons for not completing OGTTs

Women cited a variety of reasons for non‐completion of testing (Table 4). Our categorization covered the majority of reasons given for non‐completion (*n* = 204, 72.6%). Inability to tolerate the testing protocol, mainly due to nausea/vomiting associated with overnight fasting then drinking the glucose load, was the most commonly cited reason (40 of 242, 16.5% in the group with any non‐completed test). Women also cited reasons connected to their mental health and social issues for not completing testing (15% in the group with any non‐completed test). A number (37 of 242, 15.3%) of women who were initially scheduled for OGTT appointments subsequently declined testing on the basis of their general healthcare beliefs or their beliefs about pregnancy (22 of 242, 9.1%). These women had their informed refusal recorded in their medical record, and none subsequently completed testing.

**Table 4:** Reasons given by women for not completing oral glucose tolerance testing

Reasons for non‐completion	Any non‐completion (*n* = 242)	Never completed (*n* = 78)
Access issues (transport, etc.)	14 (5.8)	8 (10.3)
Unable to tolerate test protocol	40 (16.5)	16 (20.5)
Childcare issues	13 (5.4)	5 (6.4)
Social or mental health issues	37 (15.3)	17 (21.8)
Clash with other appointments, admissions	34 (14.0)	12 (15.4)
Instructions for test not followed	16 (6.6)	2 (2.6)
Unable to get convenient appointment	34 (14.0)	20 (25.6)
Declined testing after discussion	22 (9.1)	22 (28.2)
Other reasons or no reason given	71 (29.3)	22 (28.2)

Values are number (% of group total) who cited each category of reason for not completing testing. Multiple reasons were commonly cited and all reasons cited by each woman were included in the analysis. Women who never completed testing are a subset of the group with any non‐completed test.

**Figure 1:**
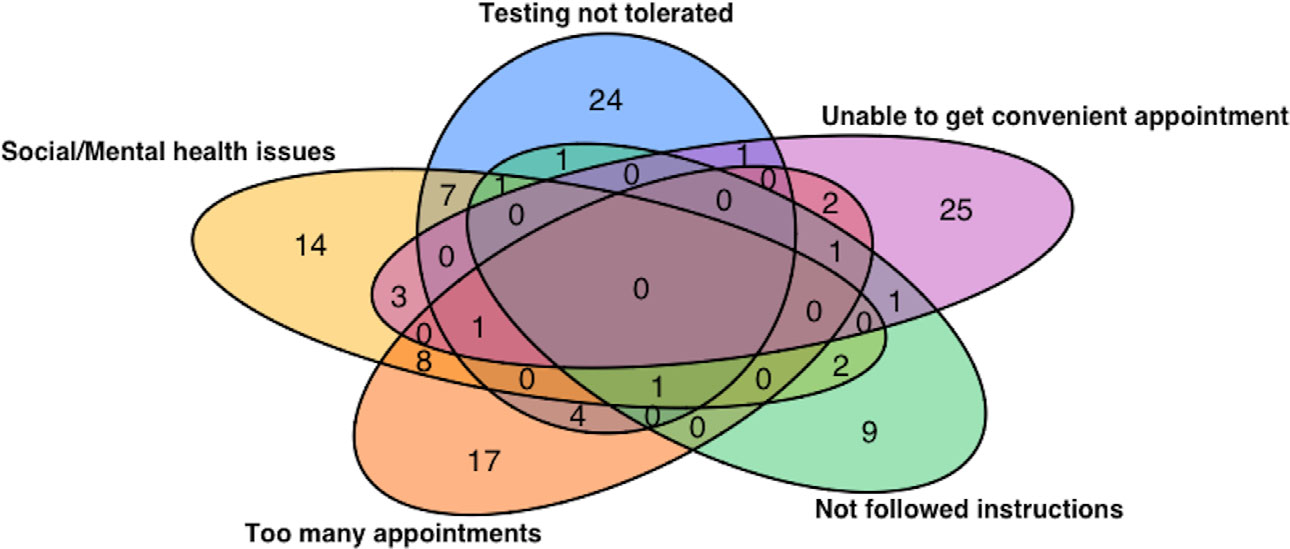
Venn diagram showing the most commonly cited reasons for non‐completion of antenatal oral glucose tolerance testing.

**Figure 2:**
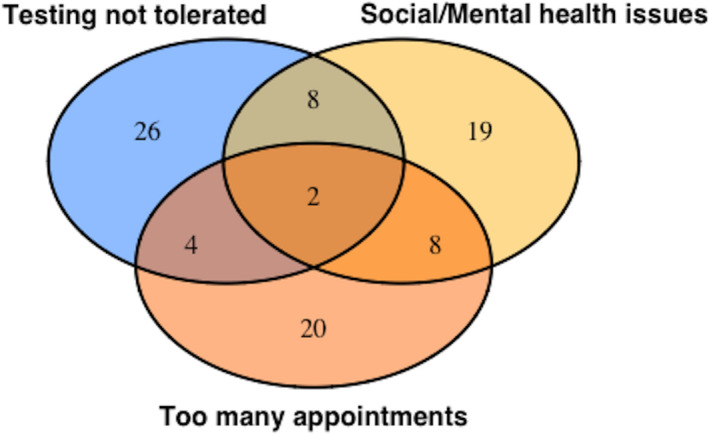
Venn diagram showing the most commonly cited triad of reasons for non‐completion, of the 22 women with overlapping factors within this triad, only eight ever completed testing.

Women commonly cited more than one factor as important in any non‐completion of testing (102 of 242, 42.1%). Multiple factors were more common in the group that never completed testing (54 of 78, 69.2%). To explore these clusters of reasons further, we visualized the overlap between the top five most commonly cited reasons (Fig. 1; excluding informed refusal, which was most commonly cited as a single reason). From further analysis of clustering, we identified a commonly cited triad of reasons (unable to tolerate test protocol, social or mental health issues, and clashes with other appointments; Fig. 2). Of the 22 women with overlapping factors within this triad, only eight ever completed testing.

## Discussion

Non‐completion of OGTTs puts women and babies at risk of delayed or missed diagnosis of GDM. We have identified key demographic factors associated with non‐completion, including younger maternal age, lower socio‐economic status, belonging to a minority ethnic group, and higher parity. Test indication was a key predictor of completion, with non‐completion less likely if women were invited due to scan findings and more likely if indications included family history or high BMI. The principal reasons for non‐completion cited by women were not being able to tolerate the test protocol, social or mental health issues, and clashes with other appointments or admissions.

Women from vulnerable (younger, lower socio‐economic status, and minority ethnic) groups were less likely to complete testing. It is known that such groups are likely to find it more difficult to access healthcare in general and to navigate systems 20, 21. Social or mental health issues were also reported as reasons for non‐completion, further demonstrating the need for increased support of vulnerable women. Although the distance women lived from the test centre was not directly correlated with non‐attendance in our cohort, a number of women cited transport and childcare issues as barriers to attendance. A more accessible alternative to an OGTT could improve test completion; for example, enabling testing in the community. An Irish trial of universal antenatal OGTT screening in primary care previously attempted to address access barriers, but found screening rates were significantly lower than in secondary care due to practical and logistical difficulties experienced by primary care providers 22. Recently, a self‐administered home OGTT, requiring women to complete finger‐pricks before and after drinking a glucose load, has been trialled and shows good agreement with laboratory results 23.

Our results also indicate that the test protocol itself presents a significant barrier to completion. In our cohort, not being able to tolerate the OGTT due to nausea was the most common reason for non‐completion. Several studies have explored substituting a glucose drink with food, e.g. ice cream, a muffin and beverage, or a specially designed breakfast 24, 25, 26. These studies all demonstrated significant correlation between the results from the standard OGTT glucose load and tests using food. Our results highlight the urgent need for further work on better‐tolerated alternative test designs. Alternative methods of diagnosis could also be considered, for example self‐monitoring of blood glucose 27, 28.

Women invited for an OGTT on the basis of a scan result were more likely to attend and there were no ‘never attenders’ with this or test results as indications. By contrast, women whose indications included family history of diabetes were significantly less likely to complete testing. Parous women were also significantly less likely to attend antenatal testing, both in our cohort and a previous study 13; however, childcare issues were only directly cited by 5.4% of women in our cohort. Women whose previous pregnancies had good outcomes or who have family experience with diabetes may perceive the diagnosis of GDM differently from primiparous women or those facing an abnormal test result. Better understanding of risk perception among women with different testing indications could help to improve engagement, and qualitative studies would be of benefit to explore this further. Women whose indication for testing was a high BMI were also less likely to attend. This may relate to risk perception, although fears of shaming or judgement could also deter women from accessing screening. Previous studies have explored these issues in women with high BMI undergoing GDM treatment, who describe feeling under surveillance and being judged as ‘good mothers’ by healthcare staff 29, 30. Clear explanations of risk factors and GDM aetiology, using sensitive and non‐judgemental language, could potentially increase test completion.

### Strengths

A large and complete contemporaneously collected data set containing detailed demographic information was available for analysis. A major strength of the study was that the barriers women faced with regards to attendance were recorded in their own words shortly after any non‐completed test by specialist midwives.

### Limitations

Although our approach gives a good balance between the narrative and quantitative aspects, further work involving in‐depth qualitative analysis would be of benefit in designing future interventions. Data on subsequent engagement with GDM treatment and on pregnancy outcomes were not available, but would be of interest in improving outcomes. The population attending our single testing centre may not reflect specific barriers faced in other pregnancy populations (e.g. we have less ethnic diversity than the UK as a whole), and thus further work is required to determine the generalizability of these findings.

## Conclusions

Based on our findings we suggest three key areas for intervention which could improve completion rates: increased support for vulnerable groups, modification of testing protocols, and improved communication regarding risk. Younger women, those from lower socio‐economic status backgrounds, minority ethnic groups, and those with mental health or social issues are likely to need additional support to navigate systems and ensure that their pregnancy healthcare needs are met.

## Funding sources

Catherine Aiken is supported by an Isaac Newton Trust [12.21(a)]/Wellcome Trust ISSF [105602/Z/14/Z]/University of Cambridge Joint Research Grant. Claire Meek is supported by the Diabetes UK Harry Keen intermediate clinical fellowship (17/0005712) and the European Foundation for the Study of Diabetes/ Novo Nordisk Foundation Futures Leader’s Award (NNF19SA058974). Juliet Usher‐Smith is supported by a Cancer Research UK Prevention Fellowship (C55650/A21464). The funders have had no role in study design, data collection, data analysis, manuscript preparation and/or publication decisions.

## Competing interests

None declared.
